# Spectral Data Collection by Dual Field-of-View System under Changing Atmospheric Conditions—A Case Study of Estimating Early Season Soybean Populations

**DOI:** 10.3390/s19030457

**Published:** 2019-01-23

**Authors:** Ittai Herrmann, Steven K. Vosberg, Philip A. Townsend, Shawn P. Conley

**Affiliations:** 1 The Robert H. Smith Institute of Plant Sciences and Genetics in Agriculture, The Hebrew University of Jerusalem, Rehovot 7610001, Israel; 2 Department of Agronomy, University of Wisconsin-Madison, 1575 Linden Drive, Madison, WI 53706, USA; ssvosberg@yahoo.com (S.K.V.); spconley@wisc.edu (S.P.C.); 3 Department of Forest & Wildlife Ecology, University of Wisconsin-Madison, 1630 Linden Drive, Madison, WI 53706, USA; ptownsend@wisc.edu

**Keywords:** hyperspectral, Piccolo dual field-of-view spectrometer, partial least squares regression (PLSR), soybean, site-specific population assessment, replanting

## Abstract

There is an increasing interest in using hyperspectral data for phenotyping and crop management while overcoming the challenge of changing atmospheric conditions. The Piccolo dual field-of-view system collects up- and downwelling radiation nearly simultaneously with one spectrometer. Such systems offer great promise for crop monitoring under highly variable atmospheric conditions. Here, the system’s utility from a tractor-mounted boom was demonstrated for a case study of estimating soybean plant populations in early vegetative stages. The Piccolo system is described and its performance under changing sky conditions are assessed for two replicates of the same experiment. Plant population assessment was estimated by partial least squares regression (PLSR) resulting in stable estimations by models calibrated and validated under sunny and cloudy or cloudy and sunny conditions, respectively. We conclude that the Piccolo system is effective for data collection under variable atmospheric conditions, and we show its feasibility of operation for precision agriculture research and potential commercial applications.

## 1. Introduction

Hyperspectral sensing is increasingly utilized in precision agriculture research [1,2]. Hyperspectral data (hundreds of narrow bands) can be analyzed as is or used to calculate vegetation indices that simplify the data but lose the depth and richness of analysis [3,4]. Hyperspectral measurements of plant canopy using the sun as the illumination source can be challenging [5], especially under changing atmospheric conditions, requiring frequent reference data collection by the same or another sensor. MacLellan and Malthus [6] presented the concept of a spectrometer with a dual field-of-view which alternately obtains upwelling (radiance) and downwelling (irradiance) measurements using a cosine response fore optic [7] in lieu of the standard white reference. MacArthur et al. [8] designed and presented the Piccolo, an operational dual field-of-view system, and in the current study, it was mounted on a tractor in soybean experimental plots.

Soybean seedling emergence can often be lower than the seeding rate. Thus, there is a need to assess plant populations in early development stages to guide replanting decisions [9]. The current methodology for population assessment generally consists of counting plants within representative quadrats and calculating the mean of these samples to extrapolate plants per unit area [10]. The variation in current methods of determining soybean populations combined with the time-sensitive importance of acquiring this information makes the automation of this task through sensing of crop reflectance an attractive case study. Hyperspectral imaging has been used for maize seedlings density assessment [11]. The objective of the current study was to explore a tractor-mounted Piccolo system performance under changing atmospheric conditions. As a case study to test the system, soybean plant population estimates were evaluated at early development stages when replanting decisions are to be made. In theory, the principle of operation of the Piccolo means measurements and analyses should be consistent regardless of atmospheric and light conditions (i.e., one predictive model incorporating all light conditions).

## 2. Materials and Methods

### 2.1. Study Area and Trial Design

**Figure 1:**
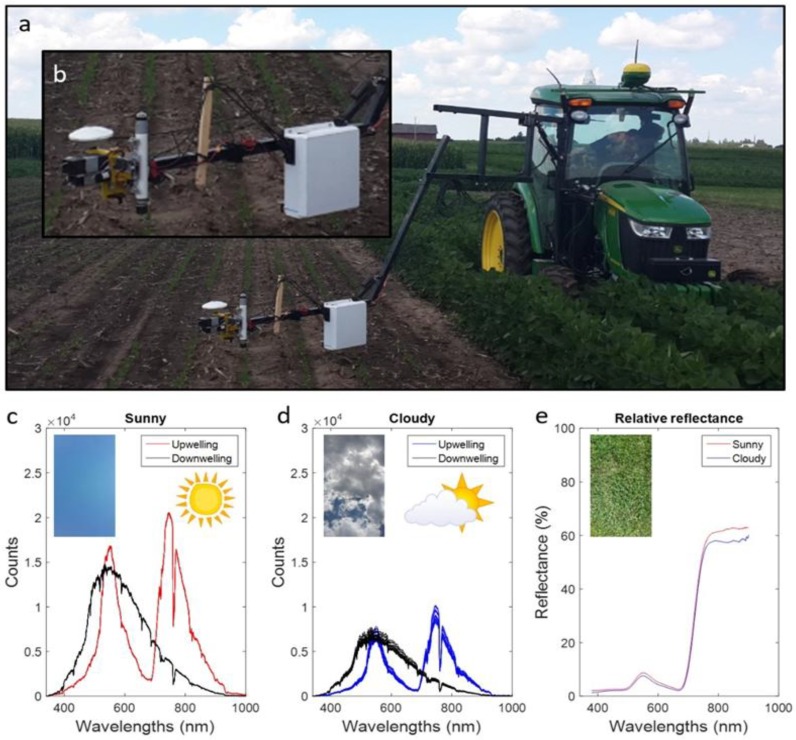
Spectral data collection under sunny and clouded atmospheric conditions. The tractor and boom deployed over soybean plants (**a**), zooming to the spectrometer box, fiber optics, shutter tube, and GPS receiver (**b**). Upwelling and downwelling raw data (counts) collected from a grassy area, obtained in sunny (**c**) and cloudy (**d**) conditions, and preprocessing resulted in relative reflectance (**e**).

Field experiments were conducted during 2016 at the University of Wisconsin Arlington Agriculture Research Station (43°18′8″ N, 89°20′8″ W). Tillage and row spacing treatments were applied for two cultivars (P28T08R—DuPont Pioneer and AG2433—Monsanto). To develop population variability, 14 seeding rates (3.7 to 51.8 seeds × 10^4^ h^−1^) were applied for each of the main effects. The plots were either four 0.76 m or six 0.38 m rows by 7.6 m in length. Driving alleys planted with soybeans were interspersed throughout the field to allow for passage of the tractor-mounted spectral system (Figure 1a). The trial was replicated, and spectral data were acquired for four early development stages (Table 1). Plant populations were determined (Table 1) per plot by counting the number of plants in 1.5 m of the center rows. The data collection dates were categorized as sunny or cloudy based on the atmospheric conditions (Table 1). Sunny was defined as clear skies and cloudy ranged from scattered to full cloud cover.

### 2.2. Spectral Data Collection

Canopy spectra of soybean seedlings were obtained using the Piccolo hyperspectral system equipped with a Flame (Ocean Optics, Inc., Dunedin, FL, USA) spectrometer with a spectral range of 340 to 1022 nm and an optical resolution of 1.33 nm full-width half-maximum interpolated to 1 nm spacing. The Piccolo system was tractor-mounted by a hydraulically actuated boom (Figure 1a). The downwelling fiber viewed the sky through a cosine corrected polytetrafluoroethylene fore-optic, while the upwelling bare fiber viewed the target with 25° view angle, both protected by an optical glass domes (Figure 1b) and fitted with shutters. A Raspberry Pi (Raspberry Pi Foundation, Cambridge, UK) single-board computer synchronized shutter activity and integration times [8]. The GPS (Geo 7x, Trimble Inc, Sunnyvale, CA, USA) data were differentially corrected in Pathfinder software (Trimble Inc, Sunnyvale, CA, USA) and the estimated accuracy ranged from 0.05 to 0.15 m for 99.96% of the corrected positions. The GPS data were used to relate spectral data to physical plot locations. The upwelling fiber was centered on a soybean row ~0.60 m above the ground, resulting in a ~0.13 m radius field of view at ground level. Spectral data were collected within two hours of solar noon, and two passes per plot per collection date allowed data collection from the two center rows. The integration times were set to maximize unsaturated signal, with values of 4–6 and 13–15 ms for downwelling and upwelling, respectively. One spectral sample was computed from a four-measurement sequence: (1) upwelling dark current; (2) upwelling target measurement; (3) downwelling target; and (4) downwelling dark current. The spectrometer obtained upwelling and downwelling data almost simultaneously (~0.6 s) using two fiber optics (400 μm and 600 μm, respectively) completely covering the 1000 μm long (25 μm wide) spectrometer slit. Such rapid measurements by the same spectrometer from both fore optics minimized the atmospheric effect on relative reflectance data (Figure 1c–e). Tractor speed was set to 0.22 m s^−1^ and actual time between the starting points of two successive spectral samples was ~2.6 s, resulting in 13 spectral samples collected per plot row.

### 2.3. Data Processing and Statistical Analysis

Relative reflectance was obtained by first subtracting dark current from upwelling radiance and downwelling irradiance, then the radiance was divided by the irradiance, while accounting for fiber diameter, integration times, and field of view (Python scripts at https://github.com/prabu-github/tracolo); the equation is presented in [13]. The 1 nm spectra were smoothed by a 2nd order polynomial and 5 nm filter [14] and averaged, resulting in relative reflectance in the range of 400 to 900 nm in 5 nm intervals averaged per plot.

The normalized difference spectral index (NDSI) [15] analysis was applied to explore the quality of correlation of all possible two-bands with the measured plant population. Partial least squares regression (PLSR) [16] a practical predictive tool for spectral reflective data, was used to estimate soybean plant populations as a function of the hyperspectral measurements. Datasets were calibrated, cross validated, and independently validated. Calibration and cross validation was done for 75% of the samples. The calibration and cross validation process was iterated 100 times for random sample distribution of calibration (75%) and cross validation (25%), resulting in 100 models. These models were independently validated for the samples left out of the calibration and cross validation process, resulting in estimated plant population values. The estimated values were averaged to obtain the mean estimated population, and the estimated vs. measured plant population values were regressed to obtain R^2^ and root mean square error (RMSE) of calibration, cross validation, and validation. The PLSR analyses and results processing were conducted in R 3.3.2 [17]. 

## 3. Results and Discussion

**Figure 2:**
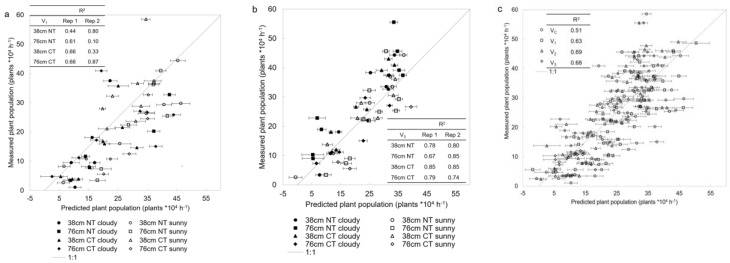
Plant population (plant × 10^4^ h^−1^) predicted by partial least squares regression (PLSR) vs. measured. Prediction of plant population for row spacing treatments, 38 and 76 cm, and till treatments NT (no till) and CT (conventional till) under sunny and cloudy conditions for validation dataset of V_1_ (vegetative) development stage (**a**) and V_3_ development stage (**b**). R^2^ values for PLSR validated by replicate 1 (cloudy) and replicate 2 (sunny) for the row spacing and till treatments are presented as well. Prediction of plant population, for development stages V_C_, V_1_, V_2_, and V_3_ by validation datasets including all treatments and both replicates together (**c**). R^2^ values for PLSR validated by development stage. Error bars are the standard deviation of 100 cross validation iterations.

Plant population predictions at V_1_ and V_3_ development stages using PLSR were obtained under sunny conditions in one trial replicate and under cloudy conditions in the other (Table 1). The quality of predictions was not affected by atmospheric conditions (Figure 2a,b), with no trends in residuals between sunny and cloudy predicted vs. measured plant populations. The later development stages showed higher R^2^ of independent validation and smaller RMSE, as expected based on the time of population count (Table 1). The V_1_ predictions were worse than V_3_, with V_1_ plant populations overestimated by both sunny and cloudy plots. This was expected since V_1_ seedlings are smaller and thus less likely to be in the Piccolo field of view. For V_3_ plots with more than ~30 plants × 10^4^ h^−1^ (hectare^−1^), there was some underestimation (Figure 2b). At this development stage, seedling foliage overlaps more than earlier stage plants such that higher populations have an increased probability of overlapping. The prediction vs. measured plant population for development stage (treatments and replicates pooled together) showed that models based on sunny and cloudy data (V_1_ and V_3_) have similar prediction qualities as those based on cloudy alone (V_2_; Figure 2c). The R^2^ and root mean square error (RMSE) of all calibration cross validation and validation PLSR models down to treatment level (32) as well as development stage models (4) and all-data-together (1) are presented in Table S1. There was no robust advantage for one of the two till treatments. The development stages as well as all-data-together models pooled different row spacing and tillage treatments; this generalization reduces the quality of the models, especially in the RMSE values. The RMSE values for the general models were more than 10 times higher than the RMSE values of the treatment-specific models. The NDSI found 565 and 710 nm in the top 10 band combinations for 15 out of the 32 datasets explored. The importance of green- and red-edge bands in maize stand counts is supported by Thorp, Steward, Kaleita, and Batchelor [11]. The NDSI_[565, 710]_ plant population estimation models further support the ability of the spectral system to provide high quality data under sunny and cloudy conditions. All NDSI_[565, 710]_ models are presented in Table S2 and the figures in Figure S1. The PLSR models resulted in better population assessment than the NDSI, showing the advantage of many narrow bands over a two bands combination.

The Piccolo system provided an efficient method for the collection of plant reflectance by eliminating the need for frequent reference standard measurements. The performance of the system was tested prior to the commencement of the current study by measuring an area of grass near solar noon under sunny and cloudy conditions. To avoid device-induced shade on the target, the fore optics were moved a few centimeters. As expected, the photon counts for downwelling and upwelling were significantly greater under sunny conditions compared to cloudy conditions (Figure 1c–e). However, the relative reflectance spectra were nearly identical in the visible and red-edge wavelengths, with slight divergence in the near infrared bands.

## 4. Conclusions

The Piccolo dual field-of-view spectrometer system was found effective at data collection under changing atmospheric conditions. This demonstrates the feasibility of its operation for precision agricultural research and potential commercial applications. For the case study of plant population assessment, (1) the best development stages for plant population assessment specifically for treatments (row spacing and tillage; Table S1) are V_2_ and V_3_, while for pooling treatments per development stage, V_1_, V_2_, and V_3_ models are of similar quality; and (2) hyperspectral data resulted in better population assessment than NDSI_[565, 710]_. Although other technologies may be suitable for crop population monitoring, once systems such as the Piccolo become part of the standard suite of crop monitoring instrumentation mounted on tractors or other agricultural implements, it will become essential to test the instrument for a range of uses. Software and hardware modifications would be required to explore aerial implementation and more efficient data collection.

## Figures and Tables

**Table 1:** Key dates for agronomical activities and spectral data collection.

Replicate 1			Replicate 2		
Date	Development Stage	Activity	Date	Development Stage	Activity
31-May-2016		Planting	15-Jul-2016	-	Planting
17-Jun-2016	V_C_	Spectra	27-Jul-2016	V_C_	Spectra
21-Jun-2016	V_1_	Spectra *	1-Aug-2016	V_1_	Spectra
26-Jun-2016	V_2_	Population count	2-Aug-2016	V_1_	Population count
27-Jun-2016	V_2_	Spectra	4-Aug-2016	V_2_	Spectra
29-Jun-2016	V_3_	Spectra	8-Aug-2016	V_3_	Spectra *

^1^ Development stages are all V (vegetative) with subscripts indicating stage of development. V_c_ indicates the cotyledon leaf stage; V_1_ indicates the first completely unrolled true leaf; V_2_ indicates the second completely unrolled true leaf; V_3_ indicates the third completely unrolled true leaf on the main stem [12]. * stands for spectral collection under sunny conditions for all data obtained this day.

## Supplementary Materials

The following are available online at http://www.mdpi.com/1424-8220/19/3/457/s1, Table S1: Results of PLSR calibration and validation plant population prediction models, Table S2: Results of NDSI calibration and validation plant population prediction models, Figure S1: Plant population (plant per h × 10^4^) predicted by NDSI vs. measured. Prediction of plant population for row spacing treatments, 38 and 76 cm, and till treatments NT (no till) and CT (conventional till) under sunny and cloudy conditions for validation dataset of V_1_ (vegetative 1) development stage (**a**) and V_3_ development stage (**b**). R^2^ values for NDSI validated by replicate 1 (cloudy) and replicate 2 (sunny) for the row spacing and till treatments are presented as well. Prediction of plant population for development stages V_C_, V_1_, V_2_, and V_3_ by validation datasets including all treatments and both replicates together (**c**). R^2^ values for NDSI validated by development stage.
