# Supplementary material for: Spectral Data Collection by Dual Field-of-View System under Changing Atmospheric Conditions—A Case Study of Estimating Early Season Soybean Populations
Source: Sensors (Basel). 2019 Jan 23;19(3):457. doi: 10.3390/s19030457 (PMC6386976; doi:10.3390/s19030457)
Supplement: Supplementary file 1 [file sensors-19-00457-s001.zip › Sup3_NDSI_Figure_S1.pdf]

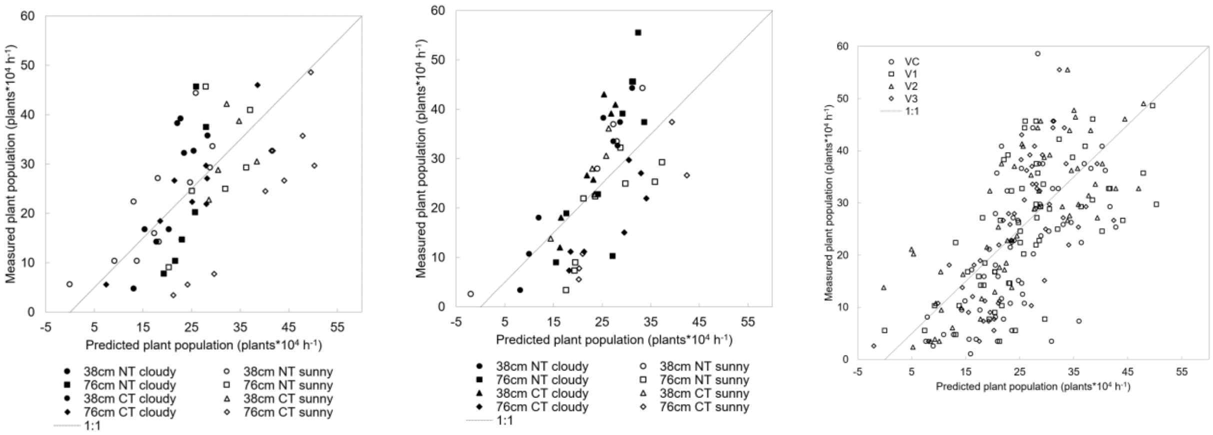

(**a**) (**b**) (**c**)

Figure S1. Plant population (plant per h × 10^4) predicted by NDSI vs. measured. Prediction of plant population for row spacing treatments, 38 and 76 cm, and till treatments NT (no till) and CT (conventional till) under sunny and cloudy conditions for validation dataset of V_1 (vegetative 1) development stage (**a**) and V_3 development stage (**b**). R^2 values for NDSI validated by replicate 1 (cloudy) and replicate 2 (sunny) for the row spacing and till treatments are presented as well. Prediction of plant population for development stages V_C, V_1, V_2, and V_3 by validation datasets including all treatments and both replicates together (**c**). R^2 values for NDSI validated by development stage.
